# Keratitis

**Published:** 1869-12

**Authors:** F. K. Bailey

**Affiliations:** Knoxville, Tenn.


### ARTICLE XLIV.

### KERATITIS.

BY F. K. BAILEY, M.D., Knoxville, Tenn.

I was called, March 31st, 1869, to go into the country two miles, to visit a woman who had been suffering from diseased eyes for some time, and had been treated by a quack pretender.

In the morning, she had taken some medicine, the effects of which alarmed the family. Found the patient a young married woman, about 22 years of age, a native of E. Tenn., and the mother of one child, about 18 months old. Temperament nervous, lymphatic, light hair and complexion. Just arousing, so as to speak, I entered the room. Found that she had taken opium. Made no further examination than to be satisfied that she would recover from the overdose, and merely learned that she had suffered from an affection of the eyes for some months.

*April 8th.* Mrs. H., above mentioned, called upon me, at my office, requesting that I should treat her case. Found her pulse feeble, and the extremities cool. Face pale; tongue clean, but flabby. Bowels not constipated, for the reason that she had taken calomel and other cathartics, with a view "to regulate the system." She had not been allowed to eat hearty food, and having no desire for any other, she had been poorly nourished, with a free secretion of milk added as a drain upon the strength. Suspected a strumous diathesis. Besides, I was informed that an older brother was nearly blind from disease of the eyes, contracted while a soldier in the late war. On examination, I found considerable thickening of the cornea, at its upper portion, in the left eye, with a general cloudy appearance, approaching opacity. The sclerotic coat slightly congested. Photophobia severe.

The right eye presented a similar appearance, but to a less degree painful, with no infiltration in the cornea. The same cloudy appearance was seen, however, as though this was only a little late in taking on a condition like the other. There was indistinctness of vision, and in cloudy weather she found it difficult to read.

Considering it a case of debility, I advised the use of the following mixture:—

℞. Syr. Sarsa. Comp., ---------------------- ℥ij.

Liquor Iodidi Ferri, ---------------------- ℥ss.

Potassii Iodidi,---------------------------- ʒij.

Sulphatis Cinchoniæ,---------------------- ℈j.

Syr. Zinziber,------------------- ------ ℥j.

M. Sig. Teaspoonful at each meal. No local applications, but pure water. To wean the child.

*15th.* Patient called. Found the appearance of the eyes unchanged, but the general health improved. To continue the mixture.

*28th.* Patient called, when I found her much improved. The opacity disappearing. Less congested appearance of the eyes. No pain, but still complains of weakness of vision and some intolerance of light. There is an inability to raise the upper lids, giving a drooping appearance to the eyes. Suspended the mixture, and prescribed as follows:—

℞. Sul. Quinine, ---------------------------- ℈j.

Ferri Iodidi, ------------------------------ ℈j.

Ext. Nucis Vomicæ, -------------------- grs. x.

M. F. Pill, No. 20. Sig.; one at each meal.

*May 25th.* Mrs. H. called, when I found all appearance of opacity gone and the sight much improved. No pain, and the action of the lids perfect. Pulse much stronger. Looking quite healthy. No appearance of menses. Complains of having ordinary headache at times, which is attributable to lingering debility.

To continue the pills, and to take two grains sub. nit. bismuth, after eating, for a slight uneasiness in the stomach, sometimes felt.

*October 14th.* Since last report, I have seen the patient occasionally, and she seems to enjoy perfect health. Her eyes are entirely well.

It will be seen that, in this case, constitutional treatment only, was employed. Not even a laxative was prescribed.

The object in reporting it was, to make it a theme of some observations upon the prevalence of scrofula in this region of country. This woman had been healthy, till, by some cause, inflammation of the eyes occurred. Instead of being confined to the conjunctiva, the cornea became affected. There was every reason to believe that, if medication of an appropriate character had not been adopted, destruction of tissue would have resulted, or deposition of lymph in the laminæ of the cornea, causing total loss of sight. The constant drain upon the strength, caused by secretion of milk, and the antiphlogistic course followed by the first doctor, reduced her very rapidly. The rapidity with which the morbid deposition in the cornea was removed, after commencing tonic treatment, shows how important it is to guard the system against every cause which tends to disorganization of tissue.

The brother above mentioned came to me in September last, for biennial examination, as a pensioner. He is laboring under opacity of the cornea of both eyes. At some points, the infiltration is so thick as to cause protrusion. Vision in one eye is totally destroyed and nearly so in the other. The disease commenced with ordinary conjunctivitis, but being of a strumous diathesis, the fibrous tissue became affected, almost immediately.

With a view of improving the general health and the prevention of destructive ulceration, more than any hope of restoring the eyesight, I prescribed this man as follows:—

℞. Liquor Iod. Ferri,-------------------------- ℥j.

Iod. Potassii,------------------------------ ℥ss.

Aqua Puræ, } āā,---------------------- ℥ij.

Syr. Zinzibins, }

Essence Cinnamon,------------------------ ℥j.

M. Sig. Teaspoonful *mane et nocte.* Also—

℞. Hyd. Bi-chloridi, ------------------------ grs. ij.

Sul. Quinine,----------------------------- ℈j.

Ext. Conii Mac.,------------------------- ℈j.

" Gentian, q. s. -------------------

M. F. Pill, No. 40. Sig.; one at night.

Saw him a few days since, and some improvement in the appearance of the eyes. Less pain on exposure to a strong light, and more ability to open and control the lids. During the last summer, I was consulted in the case of a young sister of the same family, but nine years of age.

She had no appearance of disease of the eyes, but there was the same drooping and uncertain look, commonly seen in feeble persons. There was increased sensibility to light. While quite a child she had rheumatism, and for five years she has had palpitation, with dyspnœa, on exertion, and constant pain in the cardiac region. The left side of the chest is much the fullest, and all the physical signs of hypertrophy. There was also fulness of the abdomen, with every indication of enlargement of the mesenteric glands. Anthelmentics being always in order here among the children, gave santonine and hyd. cum. creta, with the effect to bring away a number of round worms. Then followed with iod. potassii, iod. of iron, and sul. quinine, with the effect to show a decided improvement in the general health. She is now able to attend school, and, with the exception of the cardiac troubles and the tumid abdomen, is well. Nothing but a judicious course of tonics will prevent her from filling an early grave, from strumous disease of the mesentery. Should inflammation occur in the eyes, from any cause, disease of the cornea would, doubtless, result at once. This family are by no means of the class known as "*poor whites.*" They suffered from privations during the war, but since 1864, have lived comfortably on a farm. If, then, we find a tendency to struma in a family such as this, what may we not expect to see in that class who subsist mostly upon "*hog and hominy,*" and destitute of good shelter, as well as of proper food, are exposed to the numerous causes of disease. But I have extended this communication further than was first anticipated.

